# Differential MicroRNA Analyses of *Burkholderia pseudomallei*- and *Francisella tularensis*-Exposed hPBMCs Reveal Potential Biomarkers

**DOI:** 10.1155/2017/6489383

**Published:** 2017-07-16

**Authors:** Regina Z. Cer, J. Enrique Herrera-Galeano, Kenneth G. Frey, Kevin L. Schully, Truong V. Luu, John Pesce, Vishwesh P. Mokashi, Andrea M. Keane-Myers, Kimberly A. Bishop-Lilly

**Affiliations:** ^1^Genomics and Bioinformatics Department, Biological Defense Research Directorate, Naval Medical Research Center, Frederick, MD, USA; ^2^Henry M. Jackson Foundation for the Advancement of Military Medicine, Bethesda, MD, USA; ^3^KCE Services and Consulting LLC, Columbia, MD, USA; ^4^Division of Microbiology and Infectious Diseases, National Institute of Allergy and Infectious Diseases, Bethesda, MD, USA; ^5^Navy Drug Screening Laboratory, Jacksonville, FL, USA; ^6^Immunology, National Institute of Health, Bethesda, MD, USA

## Abstract

Increasing evidence that microRNAs (miRNAs) play important roles in the immune response against infectious agents suggests that miRNA might be exploitable as signatures of exposure to specific infectious agents. In order to identify potential early miRNA biomarkers of bacterial infections, human peripheral blood mononuclear cells (hPBMCs) were exposed to two select agents, *Burkholderia pseudomallei* K96243 and *Francisella tularensis* SHU S4, as well as to the nonpathogenic control *Escherichia coli* DH5*α*. RNA samples were harvested at three early time points, 30, 60, and 120 minutes postexposure, then sequenced. RNAseq analyses identified 87 miRNAs to be differentially expressed (DE) in a linear fashion. Of these, 31 miRNAs were tested using the miScript miRNA qPCR assay. Through RNAseq identification and qPCR validation, we identified differentially expressed miRNA species that may be involved in the early response to bacterial infections. Based upon its upregulation at early time points postexposure in two different individuals, hsa-mir-30c-5p is a miRNA species that could be studied further as a potential biomarker for exposure to these gram-negative intracellular pathogens. Gene ontology functional analyses demonstrated that programmed cell death is the first ranking biological process associated with miRNAs that are upregulated in *F. tularensis*-exposed hPBMCs.

## 1. Introduction

The National Institute of Allergy and Infectious Diseases (NIAID), the US Department of Agriculture (USDA), and the Centers for Disease Control and Prevention (CDC) assess and classify pathogens into different categories based on potential utilization as biological warfare agents (BWA). For instance, due to the risk of deliberate misuse and potential for mass casualties or devastating effects to the economy, critical infrastructure, or public confidence, the CDC has classified *Francisella tularensis* and *Burkholderia pseudomallei* as Tier 1 select agents. Generally speaking, the ability to appropriately respond to a biological attack depends first on rapid detection of the event. A number of techniques have previously been developed and evaluated to quickly detect pathogens in the environment as well as in humans suspected to have been exposed. For instance, nucleic acid-based assays can be used for the detection and identification of microorganisms. Examples of these techniques include real-time polymerase chain reaction (RT-PCR), microbial 16S ribosomal RNA gene sequencing, amplified-fragment length polymorphism polymerase chain reaction (AFLP-PCR), and, more recently, repetitive element polymerase chain reaction (REP-PCR) DNA fingerprinting [1]. These assays may require multiplexing in order to distinguish biological warfare agents (BWA) from near-neighbor species. For example, a quadruplex RT-PCR assay is required for the differentiation of *Yersinia pestis* from *Y. pseudotuberculosis* [2].

In addition to nucleic acid-based assays, other types of assays such as immunological and microbiological methods are available for detection of BWA. In the case of the prototypic BWA, *Bacillus anthracis*, numerous detection assays have been explored or employed—from conventional microbiological methods, immunoassays based on surface antigens and antibodies, and PCR assays based on amplification of unique regions of DNA to electrochemiluminescent assays based on ligands such as aptamers and phage display-derived peptides [3]. However, there is no single detection or diagnostic assay that would serve to identify individuals who had been exposed to a pathogen within hours of exposure and prior to onset of symptoms. Such an assay could help to rapidly identify specific populations or individuals who have been exposed as well as the agent to which those individuals were exposed. This information could drastically reduce the time to identify an effective treatment and thereby increase positive clinical outcomes.

Currently, host transcription profiles are being explored as biomarkers for infectious diseases. Ex vivo studies have provided much needed insight into the host transcriptional response to a variety of pathogens including viral [4], bacterial [5], and fungal [6] infectious agents. These studies have identified unique patterns in transcriptomic profiles to infectious agents, suggesting that transcriptome biomarkers could be a useful diagnostic tool for infectious diseases including *B. pseudomallei* [7]. Here, we explore how microRNA expression may change in response to exposure to BWA. MicroRNAs, also known as miRNAs, are highly conserved, 19–22-nucleotide-long, single-stranded, noncoding RNA (ncRNA) sequences so far mainly found in eukaryotes and viruses. miRNA research is a very active area of study, and these ncRNAs have been implicated in a wide range of physiological as well as pathological processes, including inflammatory responses, apoptosis, growth, cancer, and neurodegenerative and cardiovascular diseases [8–14]. Particularly, there is increasing evidence that miRNAs play an important role in the immune response against infectious agents, including but not limited to, *Helicobacter pylori* [15], *Listeria monocytogenes* [16], *Actinobacillus pleuropneumoniae* [17], and *Mycobacterium avium* [18]. For instance, it is well known that expression of miRNAs such as miR-155, miR-146, miR-125, let-7, and miR-21 is commonly altered during bacterial infections and contributes to immune responses aimed at protecting the organism against overwhelming inflammation [19–21]. Despite these findings, our understanding of expression patterns under normal conditions and the regulatory role of miRNAs following bacterial infections is still very limited.

To investigate the temporal changes of miRNA expression in the host cell following exposure to BWA, three different bacterial strains were used: *B. pseudomallei* K96243, *F. tularensis* SHU S4, and *Escherichia coli* DH5α as a “negative control.” While all three are gram-negative, only *B. pseudomallei* and *F. tularensis* are intracellular and pathogenic, causing melioidosis and tularemia, respectively, which are lethal if left untreated or improperly treated. Early time points, 30, 60, and 120 minutes postexposure, were investigated using the RNAseq method in order to generate a candidate list of biomarkers for BWA exposure. Following RNAseq analysis, a subset of miRNA species were chosen for validation of expression profiles using a custom qPCR array. Finally, target genes for differentially expressed miRNAs were functionally characterized using gene ontologies. We present here our findings that hsa-miR-30c-5p is a potential biomarker for infection by the gram-negative pathogens *B. pseudomallei* and *F. tularensis* and that programmed cell death-related genes were the largest constituent of genes predicted to be regulated by differentially expressed miRNAs at early time points in *F. tularensis* infection of hPBMCs.

## 2. Materials and Methods

### 2.1. Exposure of hPBMCs to *E. coli*, *B. pseudomallei*, and *F. tularensis*

Human peripheral blood mononuclear cells (hPBMCs) from two different donors: (i) a 53-year-old Caucasian female (Lot number 1F3884) for the RNAseq experiment and (ii) a 38-year-old Caucasian male (Lot number 2F3400) for the qPCR experiment, were obtained from Lonza (Walkersville, MD, USA). Cells were thawed according to the manufacturer's instructions, counted, and dispensed to 24-well tissue culture plates at ~3 × 10^6^ hPBMCs per well. The cells were grown overnight in LGM-3 growth medium (Lonza) at 37°C in 5% CO_2_. Bacterial strains *B. pseudomallei* K96243 and *F. tularensis* SHU S4 were obtained from BEI Resources (Manassas, VA, USA) and *E. coli* DH5*α* from Invitrogen (Carlsbad, California, USA). All strains were stored in single-use aliquots in 20% glycerol at −80°C prior to use. At the time of use, a single aliquot was thawed and used to inoculate 25 ml of appropriate liquid medium. *B. pseudomallei* and *E. coli* were grown overnight in Luria broth (LB), whereas *F. tularensis* was grown in tryptic soy broth (TSB) supplemented with 0.1% cysteine for 48 hours. All cultures were incubated at 37°C with vigorous shaking. Prior to exposure, hPBMCs were washed three times with fresh medium [RPMI 1640 + GlutaMAX with L-glutamine (5 ml/500 ml), 10% heat-inactivated Hyclone FBS (56° for 30+ min, 50 ml/500 ml), and 0.1% 2-mercaptoethanol (0.5 ml/500 ml)]. Using a multiplicity of infection (MOI) of approximately one, 3 × 10^6^ hPBMCs per duplicate well were exposed to *E. coli* DH5*α* (4.3 × 10^6^ CFU), *B. pseudomallei* K96243 (4.8 × 10^6^ CFU), or *F. tularensis* SHU S4 (4.2 × 10^6^ CFU). Following the addition of inoculum, the plates were centrifuged at 200 ×g for five minutes and this was designated as time zero (t0). Individual plates were prepared for three exposure intervals (30, 60, 120 minutes), and unexposed hPBMCs were included as a negative control. The plates were incubated at 37°C in an atmosphere of 5% CO_2_. All infections were conducted at biosafety level 3 (BSL-3).

### 2.2. RNA Extraction and RNA Sequencing

Prior to bacterial exposure (t0) and at each experimental time point, one plate was removed from incubation, the medium was pipetted off, and 1 ml of TRIzol^®^ (Life Technologies; Grand Island, NY, USA) was added to lyse the cells and stabilize the RNA. Sterility was confirmed according to internal protocols, and RNA was isolated according to the manufacturer's instructions. The RNA purity and quality were checked using the Agilent Bioanalyzer RNA chip (Agilent Technologies, Santa Clara, CA, USA). As per the Illumina TruSeq™ Small RNA protocol, adapter ligation, reverse transcription, PCR amplification, and pooled gel purification steps were performed to generate a small RNA library product. The libraries were sequenced on the Illumina MiSeq System using MiSeq Reagent Kit v2 (2 × 51 bp read length) using one full run per sample. As an internal positive control for alignment calculations and quantification efficiency, 15% PhiX Control v3 was spiked in.

### 2.3. RNAseq Mapping and Identification of miRNAs

Raw sequences from FASTQ files generated by the MiSeq sequencer were checked for quality using FastQC [22] and processed using several packages included in Consensus Assessment of Sequence And Variation (CASAVA v 1.8.2, Illumina) (Figure S1 available online at https://doi.org/10.1155/2017/6489383). The artificially introduced RNA 3′ adapter (RA3) with oligonucleotide sequence, TGGAATTCTCGGGTGCCAAGGC, from a TruSeq Small RNA Sample Prep Kit (Illumina) was removed using *trimmer* v3.0. Posttrimmed reads which were at least 15 base pairs (bp) in length were aligned using ELAND (Efficient Large-Scale Alignment of Nucleotide Databases) against contaminants. The contaminants screened were mitochondrial DNA, 5S ribosomal RNA, adapter, poly(A), poly(C), and enterobacteria phage phiX174 sequences from Illumina iGenome, as well as human small nucleolar RNA (snoRNA), long intergenic noncoding RNA (lincRNA), and small nuclear RNA (snRNA) from Ensembl release 69. The remaining reads which did not match to these contaminants were then aligned to human mature miRNA from the miRNA database [23, 24] (Release 19) allowing up to 2 nucleotide (nt) mismatches. These analyses were performed on duplicates of each sample at each time point.

### 2.4. Differential Expression (DE) Analysis of RNAseq Data

Replicates were assessed for correlation using the Pearson method. A time series matrix of raw counts from different time points in ascending order (t_30_, t_60_, and t_120_) was created (Supplementary Data 1), and the miRNA Temporal Analyzer (mirnaTA) [25] was used to identify DE miRNAs.

### 2.5. qPCR Confirmatory Analysis

A subset of miRNAs that were identified as differentially expressed with statistical significance (*P* < 0.05) were chosen for further investigation using a custom miScript PCR assay (Qiagen, USA). 3 reverse transcription controls (miRTC), 3 positive PCR controls (PPC), 6 housekeeping genes (SNORD95, SNORD68, SNORD96A, SNORD61, SNORD72, and RNU6-2), and 5 assay negative controls (miRNAs which were not detected (i.e., zero count) in the RNAseq reads) were included in the assay. Using total RNA as the starting material for cDNA synthesis, qRT-PCR was performed using this custom-formatted miScript SYBR Green PCR System. Quantitative RT-PCR of miRNA expression was performed in triplicates for each time point for each experiment, and the corresponding threshold cycle (C_t_) values were collected. Three housekeeping genes with the lowest standard deviation were selected for normalization [26]. The threshold cycle (C_t_) values from qPCR were analyzed using the RT^2^ Profiler PCR Array data analysis tool (Qiagen) to calculate ΔΔC_t_-based fold changes.

### 2.6. Correlation between RNAseq and qPCR Analyses

For each group of bacterial-exposed hPBMCs, correlation coefficients between the RNAseq data and the qRT-PCR data were calculated using the Pearson method. The expression data of miRNAs with *r* > 0.95 were visualized as 2D graphs. PermutMatrix [27] was also utilized to view expression data using its default parameters: Euclidean distance dissimilarity, McQuitty's method (WPGMA) hierarchical clustering, and multiple-fragment (MF) heuristic seriation rule.

### 2.7. Functional Annotation of the Targets of the DE miRNA Species

#### 2.7.1. Gene Target Prediction Using mirDB

For each organism, DE miRNAs were categorized as upregulated or downregulated. Then, the names of miRNAs within each category were submitted to miRDB target mining [28] to search miRNAs for gene targets. A relatively stringent parameter setting was used; the search was restricted to only a collection of 654 functional human miRNAs instead of a total of 2588 human miRNAs available, and gene targets with more than 80 target prediction score, instead of the default score of 60, were included. In addition, only miRNAs with fewer than 500 predicted targets in the genome were included instead of those with default 800 targets.

#### 2.7.2. Gene Ontology Using DAVID

The gene target predictions obtained from miRDB search performed in the above step were downloaded, and the resulting Entrez gene IDs were submitted to the Database for Annotation, Visualization and Integrated Discovery (DAVID) [29] with medium stringency to *Homo sapiens*. Gene ontology results were further analyzed for biological process (GOTERM_BP_FAT), cellular component (GOTERM_CC_FAT), and molecular function (GOTERM_MF_FAT). The GO terms produced were too many to be plotted on a single graph, and therefore, they were consolidated into more general terms. For instance, terms such as “positive regulation of x,” “negative regulation of x,” “regulation of x,” and “x” were consolidated into one term “x_related” (Supplementary Table S10 and S11). The number of genes in each GO term category was then counted using an in-house Perl script and visualized on the graph using R utilities. GO functions with at least five genes (10 genes in *E. coli*) falling into a given category were included.

## 3. Results

### 3.1. Quality Control Shows Good Sequencing

Most transcripts were evaluated to be between 20 and 30 bp indicating that most of the RNA species sequenced were small RNAs such as miRNAs (Figure S2). FastQC analyses showed that nearly all bases had very high-quality scores of >Q30 which is equivalent to base call accuracy of 99.9% or higher (Figure S3).

### 3.2. 87 miRNA Species Displayed Linear Differential Expression in RNAseq Data

**Figure 1:**
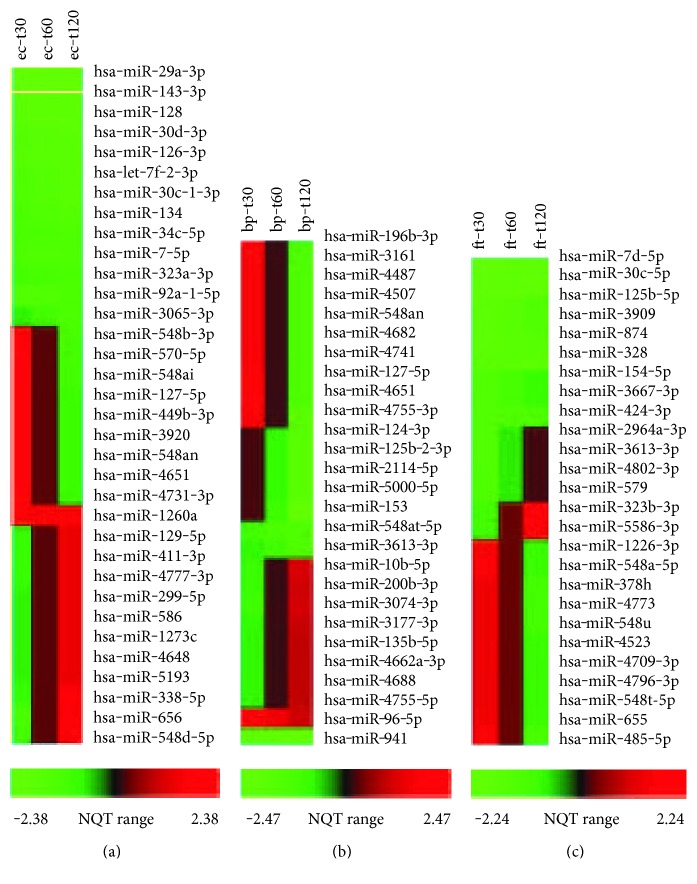
Heatmap of 87 DE miRNAs which passed a first round screening test from RNAseq analysis. (a) 34 in *E. coli*- (ec-) exposed hPBMCs, (b) 27 in *B. pseudomallei*- (bp-) exposed hPBMCs, and (c) 26 in *F. tularensis*- (ft-) exposed hPBMCs. For each organism, the three time points (t30, t60, and t120) are shown from left to right. The *x*-axis indicates the range of normal quantile transformed (NQT) data.

Posttrimmed sequencing reads (≥15 bp) were aligned to mature miRNAs in the miRBase database with up to two nucleotide mismatches allowed. Reads from *E. coli*-exposed hPBMCs matched to 569 mature miRNA species, those from *B. pseudomallei*-exposed hPBMCs to 649, and those from *F. tularensis*-exposed hPBMCs to 526, respectively. The Pearson method showed that the duplicates had very high correlation coefficient values, *r* (Figure S4 and Table S1). Normalization and analysis of the raw miRNA counts at each time point were performed using mirnaTA. A total of 87 human mature miRNA species displayed differential expression with statistical significance (*P* < 0.05). Specifically, in *E. coli*-exposed hPBMCs, there were 34 significant DE miRNAs, of which 62% displayed downregulation while 38% displayed upregulation overtime (Figure 1(a); Table S2). In *B. pseudomallei*-exposed hPBMCs, there were 27 significant DE miRNAs, of which 70% displayed downregulation while 30% displayed upregulation overtime (Figure 1(b); Table S3). In contrast to *E. coli* and *B. pseudomallei*, both of which caused overall decreased expression of a variety of human miRNA species, exposure to *F. tularensis* resulted in a more balanced change in expression profiles, such that out of 26 significantly DE miRNAs, 46% displayed downregulation and 54% displayed upregulation overtime (Figure 1(c); Table S4).

### 3.3. qPCR Assay Confirms hsa-miR-30c-5p

In order to confirm the DE patterns observed in the RNAseq data, a customized qRT-PCR array was designed. Specifically, 3, 10, and 18 miRNAs from *E. coli-*, *B. pseudomallei*-, and *F. tularensis*-exposed hPBMCs, respectively (Table S5), were chosen for further investigation. In other words, due to space limitation on the miScript plate, only 36% of the miRNA species identified in the RNAseq phase of the study (31 out of 87) were chosen for confirmation. The overall objective was to identify candidate miRNA species that could serve as early biomarkers of exposure. We presumed that a biomarker that increases in expression upon exposure would ultimately make a better target for an assay; therefore, all the miRNAs displaying increased expression in *B. pseudomallei*- and *F. tularensis*-exposed hPBMCs were chosen to be included in the PCR assay.

**Figure 2:**
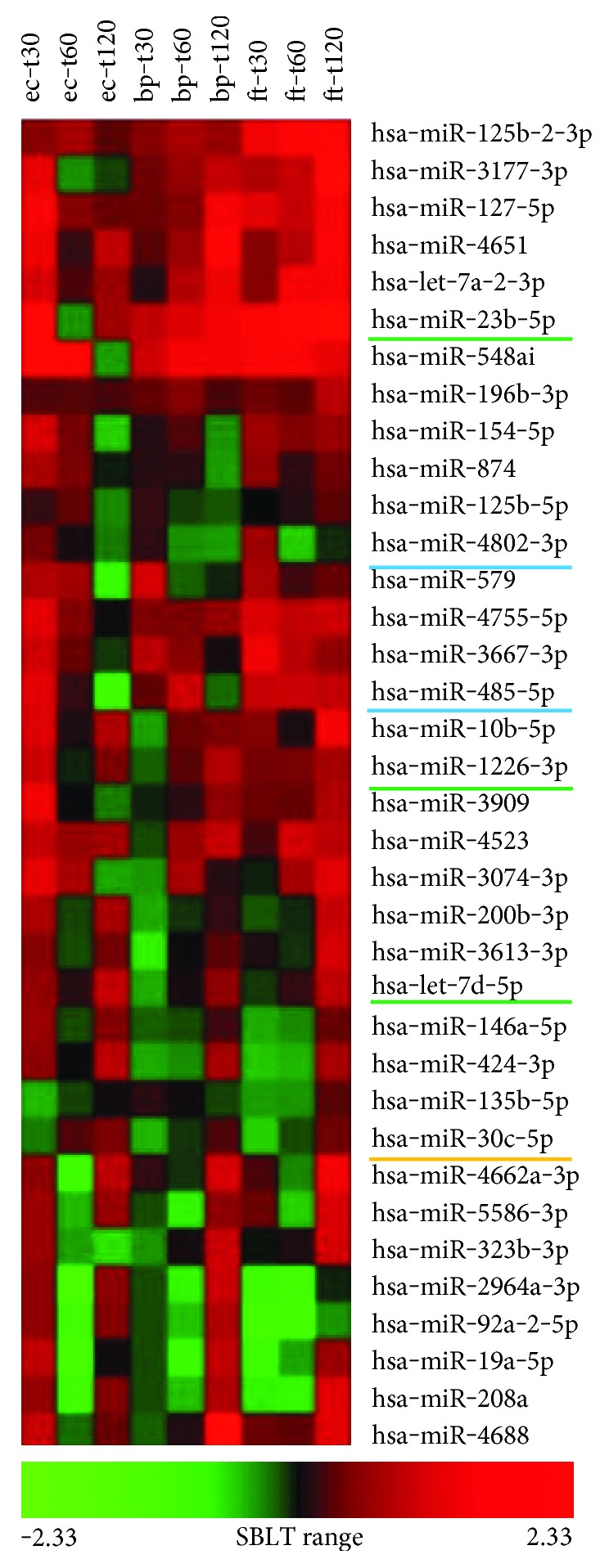
Heatmap of 36 miRNA species tested in the miScript qPCR assay. The qPCR fold changes were plotted for three time points (t30, t60, and t120) for *E. coli*- (ec-), *B. pseudomallei*- (bp-), and *F. tularensis*- (ft-) exposed hPBMCs. The miRNA species that show either decreasing or increasing expression with statistical significance (*P* < 0.05) are as follows: in *E. coli*-exposed hPBMCs, hsa-miR-485-5p and hsa-miR-4802-3p were downregulated (underlined blue); in *B. pseudomallei*-exposed hPBMCs, hsa-let-7d-5p, hsa-miR-1226-3p, and hsa-miR-23b-5p were upregulated (underlined green); in both *B. pseudomallei*- and *F. tularensis*-exposed hPBMCs, hsa-miR-30c-5p displayed upregulation (underlined gold).

**Figure 3:**
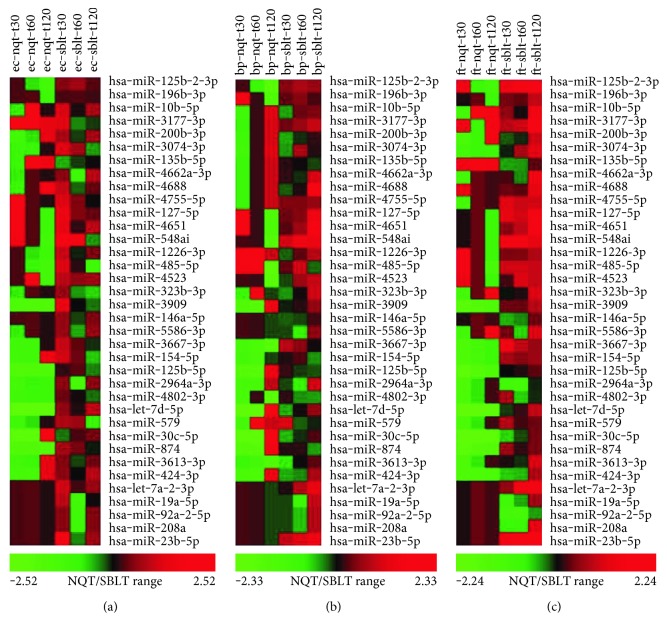
Heatmap of RNAseq (NQT) versus qPCR (SBLT) data for 36 miRNA tested in miScript qPCR. Here, RNAseq (NQT) data is shown in the first three columns, and qPCR (SBLT) data are shown in the last three columns for each experiment. The miRNAs with a correlation coefficient *r* > 0.95 are as follows: (a) *E. coli*- (ec-) exposed hPBMCs: hsa-miR-4755-5p; (b) *B. pseudomallei*- (bp-) exposed hPBMCs: hsa-miR-3177-3p, hsa-miR-200b-3p, hsa-miR-3667-3p, and hsa-miR-424-3p; (c) *F. tularensis*- (ft-) exposed hPBMCs: hsa-miR-200b-3p, hsa-miR-548ai, hsa-miR-125b-5p, hsa-let-7d-5p, and hsa-miR-30c-5p.

For qPCR, the hPBMCs from a second donor (Lot number 2F3400) were exposed in triplicate to the three bacterial organisms and harvested at the three time points as performed in RNAseq and RNA was extracted. For normalization, the three HKGs with the lowest standard deviation, SNORD95, SNORD68, and SNORD96A, were selected (Table S6). The threshold cycle C_t_ values (Supplementary Table S7) were converted using log transformation, and the linear regression model was applied to calculate all the *P* values for each miRNA fold regulation over increasing exposure time (Figure 2) (Supplementary Table S8). The expression profile of each particular miRNA species as measured by qPCR was evaluated against its own expression profile in the RNAseq data. Of the 3 miRNA species in *E. coli*- or 18 miRNA species in *B. pseudomallei*-exposed hPBMCs that were found to be DE with statistical significance from RNAseq data, none were validated by the qPCR array as being DE after exposure to those same organisms (Table 1; Figure 3). This may be explained by the fact that there were two different donors. In order to have maximal correlation between the RNAseq and qPCR data, hPBMCs from one individual should have been used for both assays. However, we chose to test using two different individuals since a usable miRNA biomarker would have to work consistently across different genders, ages, ethnicities, and so on. Regardless, one of the 18 DE miRNA species in the RNAseq data from *F. tularensis*-exposed hPBMCs, namely, hsa-miR-30c-5p, was confirmed to have the same altered expression profile in qPCR data.  Table 2 summarizes the number of miRNA species which were DE in RNAseq, included in the qPCR array, and confirmed to be DE in both RNAseq and qPCR analyses.

Additionally, 4 miRNA species selected for their consistently increasing expression in *F. tularensis*-exposed hPBMCs from RNAseq analysis exhibited statistically significant differential expression in the qPCR array postexposure to *B. pseudomallei*. These 4 miRNAs are, in ascending *P* value order, hsa-miR-1226-3p (*P* = 0.0113), hsa-miR-23b-5p (*P* = 0.0136), hsa-let-7d-5p (*P* = 0.0175), and hsa-miR-30c-5p (*P* = 0.02408). In *E. coli*-exposed hPBMCs, two miRNA species (initially selected for their consistently increasing expression pattern in *F. tularensis*-exposed hPBMCs) were found to be DE: hsa-miR-485-5p (*P* = 0.0170) and hsa-miR-4802-3p (*P* = 0.0273).

### 3.4. A Few miRNA Species Show High Correlation between RNAseq and qPCR Analyses

**Figure 4:**
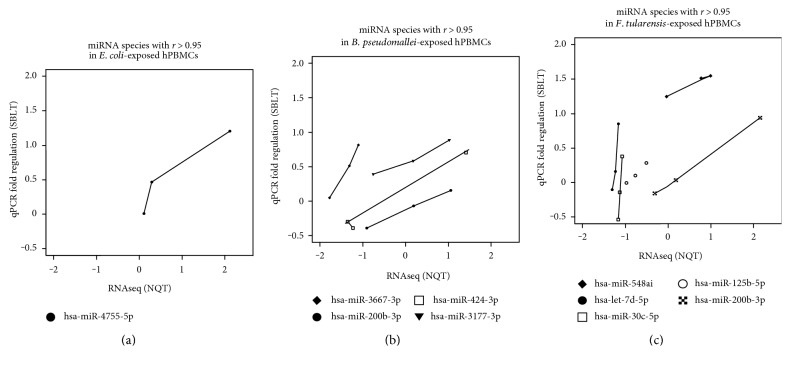
A subset of miRNA species with *r* > 0.95 between RNAseq (NQT) and qPCR (SBLT) fold changes. (a) *E. coli*-exposed hPBMCs: hsa-miR-4755 (*P* = 0.1973); (b) *B. pseudomallei*-exposed hPBMCs: hsa-miR-3177-3p, hsa-miR-200b-3p (*P* = 0.0275), hsa-miR-3667-3p (*P* = 0.0602), and hsa-miR-424-3p (*P* = 0.0670); (c) *F. tularensis*-exposed hPBMCs: hsa-miR-200b-3p (*P* = 0.0160), hsa-miR-548ai (*P* = 0.0489), hsa-miR-125b-5p (*P* = 0.0638), hsa-let-7d-5p (*P* = 0.1788), and hsa-miR-30c-5p (*P* = 0.0274).

In order to examine the overall correlation between RNAseq data and qPCR data collected, RNAseq data were plotted against qRT-PCR data for all the 31 miRNA species included in the miScript array for each set of bacterial-exposed hPBMCs. Subsequently, the miRNAs, with correlation coefficient, *r* > 0.95, were selected and plotted in a separate graph (Figure 4). In *E. coli*-exposed hPBMCs, hsa-miR-4755-5p displayed a similar expression pattern in both RNAseq and qRT-PCR (*r* > 0.95). However, the *P* value of 0.1973 did not pass a significance threshold value of 0.05 (Figure 4(a)). In *B. pseudomallei*-exposed hPBMCs, a total of four miRNAs, hsa-miR-3177-3p, hsa-miR-200b-3p, hsa-miR-3667-3p, and hsa-miR-424-3p, displayed similar expression in both RNAseq and qRT-PCR. Of these, hsa-miR-200b-3p was the only one found to be statistically significant with *P* = 0.0275 (Figure 4(b)). In *F. tularensis*-exposed hPBMCs, five miRNAs were found to have an *r* > 0.95, namely, hsa-miR-200b-3p, hsa-miR-548ai, hsa-miR-125b-5p, hsa-let-7d-5p, and hsa-miR-30c-5p. Of these, three miRNA species hsa-miR-200b-3p (*P* = 0.0160), hsa-miR-548ai (*P* = 0.0489), and hsa-miR-30c-5p (*P* = 0.0274) met the statistical significance threshold (Figure 4(c)).

### 3.5. Multiple Testing

Since RNAseq and qRT-PCR were independent technical procedures, a combined *P* value was obtained by multiplying the *P* value of RNAseq by that of qRT-PCR data. In addition, a multiple testing correction was applied [30]. That is, since 36 miRNA species were applied in the qRT-PCR plate and 5 of them were “negative controls,” a factor of 31 was used for further multiplication. Even after this correction for multiple testing, upregulation of hsa-miR-30c-5p in *F. tularensis*-exposed hPBMCs was statistically significant (Table 3).

### 3.6. Functional Annotation of the Targets of DE miRNA Species

**Figure 5:**
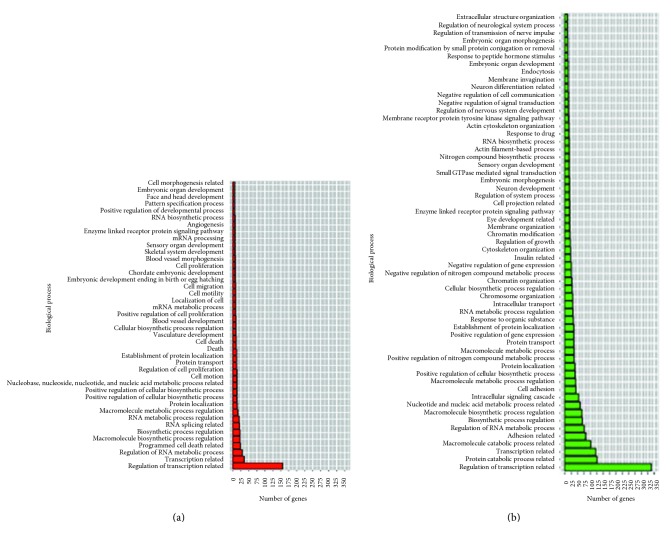
GO terms found for gene targets of differentially expressed miRNAs in response to *E. coli* exposure in hPBMCs. (a) Upregulated miRNAs; (b) downregulated miRNAs.

**Figure 6:**
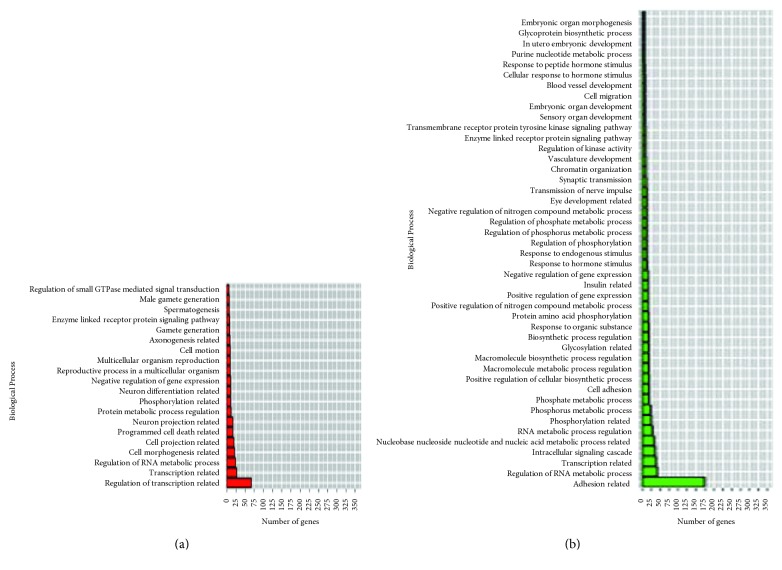
GO terms found for gene targets of differentially expressed miRNAs in response to *B. pseudomallei* exposure in hPBMCs. (a) Upregulated miRNAs; (b) downregulated miRNAs.

**Figure 7:**
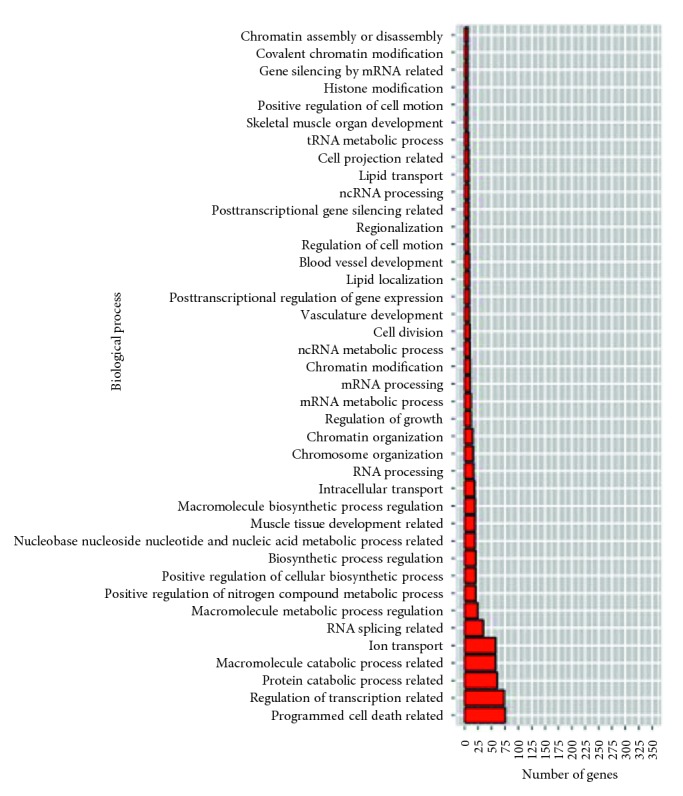
GO terms found for gene targets of upregulated miRNAs in response to *F. tularensis* exposure in hPBMCs. Programmed cell death is the top hit. Note that there are no downregulated miRNAs and thus no graphical plotting.

The miRNA species DE in each experiment were analyzed using DAVID for the discovery of their functions using GO terms (Table S9). Overall, biological process (BP) terms were called more frequently than cellular component (CC) or molecular function (MF) terms. This is probably reflected by the fact that BP GO terms are more thoroughly annotated in the literature than the others. After GO terms are grouped according to their functions and analyzed, transcription-related GO terms were found to be predominant in both downregulated and upregulated miRNAs. For the genes targeted by DE miRNAs in *F. tularensis-*exposed hPBMCs, there were no GO terms associated with downregulated miRNAs; however, for upregulated miRNAs, the “programmed cell death-related” ranked first. The same GO term was also found to be the fourth and sixth rankings in *E. coli*- and *B. pseudomallei*-exposed hPBMCs, respectively (Figures 5–7; Table 4).

## 4. Discussion

In this study, PBMCs from human donors were exposed to three different gram-negative microorganisms—*E. coli* as well as the classic BWA *B. pseudomallei* and *F. tularensis*—followed by analysis of host miRNA expression profiles at early time points. Two different methods, namely, RNAseq and qPCR, were employed. Early time points (0, 30, 60, and 120 minutes postexposure) were chosen due to our interest in discovering biomarkers that could be assayed immediately following a potential BWA exposure, to identify or triage patients prior to the onset of symptoms and thereby enable swifter initiation of the appropriate treatment(s). Although the time points chosen were very early, they were not too early for analysis of DE miRNA. A three-hour time point has previously been used in a genome-wide human miRNA stability analysis in more than ten different cell types [31] as well as in a study of the abundance changes in both stable and unstable retinal miRNAs in the mammalian light adaptation process [32]. In order to have maximal correlation between the RNAseq and qPCR data, hPBMCs from one individual should have been used for both assays. However, we chose to test using two different individuals since a usable miRNA biomarker would have to work consistently across different genders, ages, ethnicities, and so on. It was anticipated in this study that a generic response to a gram-negative pathogen might have been observed as a common signature in *E. coli*-, *B. pseudomallei*-, and *F. tularensis*-exposed cells. Whether due to differences in life style (pathogen versus nonpathogen, intracellular versus extracellular) of *E. coli* DH5*α* as opposed to the other two organisms, the early time points chosen, the small sample size, or some other unidentified factor(s), we did not observe such a phenomenon replicated among all samples and with statistical significance. However, there were some miRNA species that were commonly DE among different infection groups. For instance, hsa-miR-1226-3p, hsa-miR-23b-5p, hsa-let-7d-5p, and hsa-miR-30c-5p, which were initially identified as increasing in expression in response to *F. tularensis* exposure by RNAseq, were also found to increase in expression after exposure to *E. coli* or *B. pseudomallei*, although in the case of *E. coli* and *B. pseudomallei* exposure, this increase was detected by qPCR and not by RNAseq. Whether these variations are due to the assays employed or due to individual human genetic variation or due to some other unidentified factors, a larger number in subsequent follow-on work may help to elucidate the significance of these miRNA species in cells exposed to *B. pseudomallei*, *F. tularensis*, and *E. coli*.

Among the many miRNA species detected in this study, the one that stands out as having an unambiguously altered expression profile in response to infection is hsa-miR-30c-5p. Intracellular bacteria often survive inside their host cells by regulating the immune response to their presence; for example, *B. pseudomallei* actively downregulates the host inflammatory response through TssM-mediated inhibition of the NF-kappaB and type I IFN pathways [33, 34]. The miRNA expression data presented here may be indicative of a similar host response modulation phenomenon. The overall disparity in response to the three different organisms between the two donors' cells serves to highlight the potential importance of hsa-miR-30c-5p as a potential biomarker for further study. The RNAseq and qPCR results for hsa-miR-30c-5p were found to have a correlation coefficient of *r* > 0.95, and it also passed multiple testing. This is interesting because the hPBMCs used in RNAseq and qPCR were from two different individuals, and yet hsa-miR-30c-5p still remained statistically significant.

A recent study has shown that hsa-miR-30c, along with hsa-miR-30b, acted as a negative regulator of cell death induced by loss of attachment (anoikis) [35]. The study also showed that anoikis resistance was acquired through downregulation of caspase-3 expression by these miRNA species and that overexpression of these miRNAs resulted in a decrease in other types of caspase 3-dependent cell death. It is known that type A *F. tularensis* induces caspase-3-dependent macrophage apoptosis, resulting in the loss of potentially important innate immune responses to the pathogen [36]. Therefore, we speculate that as *F. tularensis* infection or exposure occurs, the expression of hsa-miR-30c-5p may increase to downregulate caspase-3 expression.

In addition, this miRNA, hsa-miR-30c-5p, was also found to be differentially expressed in *B. pseudomallei*-exposed hPBMCs. It has been demonstrated that apoptosis induced by *B. pseudomallei* involves a type III translocator protein, Bip B, and its interaction with caspase [37]. It is likely that similar to *F. tularensis*-exposed hPBMCs, hsa-miR-30c-5p may be upregulated to control caspase-3 expression in *B. pseudomallei*-exposed hPBMCs. From the RNAseq versus qPCR analysis, the following miRNA species were found to have a correlation coefficient > 0.95 and also met the statistical significance threshold (*P* < 0.05): in *B. pseudomallei*, hsa-miR-200b-3p, and in *F. tularensis*, hsa-miR-200b-3p, hsa-miR-548ai, and hsa-miR-30c-5p. It is remarkable that hsa-miR-200b-3p met the statistical significance threshold in this analysis for both *B. pseudomallei*- and *F. tularensis*-exposed hPBMCs.

## 5. Conclusions

Several miRNA species were identified that could be potential biomarkers for identification of bacterially infected individuals in early stages. The most interesting miRNA in this investigation was hsa-miR-30c-5p which was significant in all four different analyses in *F. tularensis-*exposed hPBMCs: RNAseq, qPCR, RNAseq versus qPCR correlation, and multiple testing. This miRNA was also found to be upregulated in *B. pseudomallei* qPCR analysis. It is our speculation that hsa-miR-30c-5p may be playing a role as a negative regulator of cell death upon infections by *F. tularensis* or *B. pseudomallei.* However, a vast amount of validation is needed to be performed before any of the proposed biomarkers could be considered potentially useful. GO term analysis revealed that programmed cell death ranked first as the biological process involved in miRNA species differentially expressed in response to *F. tularensis* exposure of hPBMCs. Even though the nonpathogenic *E. coli* DH5*α* served as a negative control, we still observed programmed cell death in the fourth place of GO functions for differentially expressed miRNA species in response to *E. coli* exposure. Similarly, *B. pseudomallei-*exposed hPBMCs also display programmed cell death in the sixth place.

## Supplementary Material

Supplementary Figure 1 | Detailed steps used for processing FASTQ files generated from MiSeq runs. Artificially introduced 3' adapter sequences were trimmed, and post-trimmed reads that were a minimum of 15 base pairs were filtered against contaminants. Reads that did not match to contaminants were screened for mature miRNA species (black box) which were further analyzed for statistical significance. Supplementary Figure 2 | RNA purity and quality. (A) The microRNA is seen between 145 nt and 160 bp on a 6% Norvex gel. The ladders used are TruSeq High Resolution Ladder and Custom Ladder (B) Agilent trace shows a good range of small RNA from 145 nt-160 nt after ligation (C) Transcript coverage shows the majority of transcript ranges between 20 and 30 bp. Supplementary Figure 3 | FASTQC analysis. An overview of the range of quality values across all bases at each position in the FastQ file. For each position, a BoxWhisker type plot is drawn. The y-axis on the graph shows the quality scores and in this run, the quality scores range from 30 to 40. Supplementary Figure 4 | Correlation plots of 2 replicates. (A) in hPBMC alone at time point 60 minutes (B) E. coli-exposed hPBMCs at time point 120 minutes. Table S1 | Correlation value (r) between duplicates. Table S2 | 34 differentially expressed miRNAs in E. coli-exposed hPBMCs RNAseq data. Table S3 | 27 differentially expressed miRNAs in B. pseudomallei-exposed hPBMCs RNAseq data. Table S4 | 26 differentially expressed miRNAs in F. tularensis-exposed hPBMCs RNAseq. Table S5 List of 31 miRNAs included in the miScript miRNA PCR assay. Table S6 | Standard deviations for six housekeeping genes. Table S7 | CT values. Table S8 | Fold regulations of miRNAs in different experiments at different time points. Table S9 | Differentialy expressed miRNAs and the number of genes involved in different GO functions. Table S10 | Proposed new broader category for each biological process GO term. Table S11 | A summary table of biological process GO terms shortened to new broader category.

## Figures and Tables

**Table 1:** Summary results of expression profiles in RNAseq versus qPCR.

miRNA name	RNA-seq expression pattern			*P* value in qPCR			Analysis result
	*E. coli*	*B. pseudomallei*	*F. tularensis*	*E. coli*	*B. pseudomallei*	*F. tularensis*	
hsa-miR-125b-2-3p		↓		0.625	0.734	0.162	Not confirmed
hsa-miR-196b-3p		↓		0.646	0.749	0.412	Not confirmed
hsa-miR-10b-5p		↑		0.770	0.272	0.525	Not confirmed
hsa-miR-3177-3p		↑		0.410	0.078	0.258	Not confirmed
hsa-miR-200b-3p		↑		0.989	0.064	0.282	Not confirmed
hsa-miR-3074-3p		↑		0.122	0.709	0.067	Not confirmed
hsa-miR-135b-5p		↑		0.145	0.058	0.308	Not confirmed
hsa-miR-4662a-3p		↑		0.891	0.454	0.516	Not confirmed
hsa-miR-4688		↑		0.654	0.272	0.367	Not confirmed
hsa-miR-4755-5p		↑		0.086	0.412	0.574	Not confirmed
hsa-miR-127-5p	↓	↓		0.262	0.275	0.625	Not confirmed
hsa-miR-4651	↓	↓		0.741	0.184	0.139	Not confirmed
hsa-miR-548ai	↓			0.310	0.385	0.408	Not confirmed
hsa-miR-1226-3p			**↓**	0.657	**0.011**	0.307	**Not confirmed in Ft but ↑ in Bp**
hsa-miR-485-5p			**↓**	**0.017**	0.713	0.599	**Not confirmed in Ft but ↓ in Ec**
hsa-miR-4523			↓	0.533	0.159	0.466	Not confirmed
hsa-miR-323b-3p			↑	0.218	0.150	0.281	Not confirmed
hsa-miR-3909			↑	0.243	0.152	0.362	Not confirmed
hsa-miR-146a-5p			↑	0.883	0.262	0.252	Not confirmed
hsa-miR-5586-3p			↑	0.989	0.650	0.709	Not confirmed
hsa-miR-3667-3p			↑	0.075	0.079	0.121	Not confirmed
hsa-miR-154-5p			↑	0.061	0.495	0.818	Not confirmed
hsa-miR-125b-5p			↑	0.483	0.242	0.102	Not confirmed
hsa-miR-2964a-3p			↑	1.000	0.554	0.379	Not confirmed
hsa-miR-4802-3p			**↑**	**0.027**	0.299	0.583	**Not confirmed in Ft but ↓ in Ec**
hsa-let-7d-5p			**↑**	0.719	**0.018**	0.164	**Not confirmed in Ft but ↑ in Bp**
hsa-miR-579			↑	0.292	0.394	0.495	Not confirmed
hsa-miR-30c-5p			**↑**	0.179	**0.024**	**0.046**	**Confirmed in Ft and also ↑ in Bp**
hsa-miR-874			↑	0.085	0.357	0.714	Not confirmed
hsa-miR-3613-3p			↑	0.893	0.155	0.423	Not confirmed
hsa-miR-424-3p			↑	0.751	0.289	0.303	Not confirmed
hsa-let-7a-2-3p	ND	ND	ND	0.642	0.057	0.311	Not confirmed
hsa-miR-19a-5p	ND	ND	ND	0.708	0.596	0.118	Not confirmed
hsa-miR-92a-2-5p	ND	ND	ND	0.973	0.509	0.362	Not confirmed
hsa-miR-208a	ND	ND	ND	0.973	0.509	0.341	Not confirmed
hsa-miR-23b-5p	ND	ND	ND	0.622	**0.014**	0.084	**Not confirmed but ↑ in Bp**

ND = not detected in RNAseq data.

**Table 2:** Summary table of the number of miRNAs differentially expressed in RNAseq, included in the qPCR assay, and confirmed in qPCR.

Sample	Number of miRNAs								
	Differentially expressed in RNAseq			Included in the qPCR assay			Confirmed in qPCR		
	Down	Up	Total	Down	Up	Total	Down	Up	Total
*E. coli*-exposed hPBMCs	21	13	34	3	0	3	0	0	0
*B. pseudomallei*-exposed hPBMCs	19	8	27	5	8	13	0	0	0
*F. tularensis*-exposed hPBMCs	12	14	26	3	15^∗^	18	1	0	1

^∗^In addition to the 14 miRNA species found to be upregulated, hsa-miR-146a-5p was also included in the qPCR assay as it is a common miRNA often found to be involved in response to bacterial infection.

**Table 3:** Multiple testing of *P* values for the 10 miRNAs which had correlation coefficient > 0.95 between RNAseq and qPCR.

	miRNA name	RNAseq	qPCR	Meta-analysis (*P* value × *P* value)	Multiple testing correction (*n* = 30^#^)
*E. coli*	hsa-miR-4755-5p	0.2837	0.0863	**0.0245**	0.7345
*B. pseudomallei*	hsa-miR-3177-3p	**0.0231**	0.0775	**0.0018**	0.0537
	hsa-miR-200b-3p	**0.0364**	0.0639	**0.0023**	0.0698
	hsa-miR-3667-3p	0.139	0.0789	**0.0110**	0.3290
	hsa-miR-424-3p	0.3557	0.2887	0.1027	3.0807
*F. tularensis*	hsa-miR-200b-3p	0.2659	0.2819	0.0750	2.2487
	hsa-miR-548ai	0.4567	0.4078	0.1862	5.5873
	hsa-miR-125b-5p	**0.0377**	0.1015	**0.0038**	0.1148
	hsa-let-7d-5p	**0.0146**	0.1642	**0.0024**	0.0718
	hsa-miR-30c-5p	**0.0189**	**0.0463**	**0.0009**	0.0263

^#^Multiplied by 30 since there were 31 miRNAs in the qPCR assay minus itself.

**Table 4:** GO terms found in the miRNAs differentially expressed in different organism-exposed samples.

GO term	*E. coli*-exposed hPBMCs	*B. pseudomallei-*exposed hPBMCs	*F. tularensis*-exposed hPBMCs
(a) Downregulated miRNAs			
Top 1	Regulation of transcription related	Regulation of transcription related	N/A
Top2	Protein catabolic process related	Adhesion related	
Top 3	Transcription related	Regulation of RNA metabolic process	
Top 4	Macromolecule catabolic process related	Transcription related	
Top 5	Adhesion related	Intracellular signaling cascade	
(b) Upregulated miRNAs			
Top 1	Regulation of transcription related	Regulation of transcription related	**Programmed cell death related**
Top2	Transcription related	Transcription related	Regulation of transcription related
Top 3	Regulation of RNA metabolic process	Regulation of RNA metabolic process	Protein catabolic process related
Top 4	**Programmed cell death related**	Cell morphogenesis related	Macromolecule catabolic process related
Top 5	Macromolecule biosynthetic process regulation	Cell projection related	Ion transport
Top 6		**Programmed cell death related**	

## Abbreviations

AFLP-PCR: Amplified-fragment length polymorphism polymerase chain reaction
BP: Biological process
BWA: Biological warfare agents
CC: Cellular component
CDC: Centers for Disease Control and Prevention
DE: Differentially expressed or differential expression
HKGs: Housekeeping genes
hPBMCs: Human peripheral blood mononuclear cells
MF: Molecular function
miRNAs: MicroRNAs
NIAID: National Institute of Allergy and Infectious Diseases
PPC: Positive PCR controls
REP-PCR: Repetitive element polymerase chain reaction
RT-PCR: Real-time polymerase chain reaction
RTC: Reverse transcription controls.

## Additional Points

*Availability of Supporting Data*. RNAseq data sets were deposited in the Short Read Archive (SRA) under the following IDs: unexposed [SRX1421931, SRX1422013, SRX1422015, and SRX1422194], *E. coli*-exposed [SRX426123, SRX426124, and SRX426125], *B. pseudomallei*-exposed [SRX424863, SRX426391, and SRX426392], and *F. tularensis*-exposed [SRX426475, SRX42647, and SRX426478].
