# Supplementary material for: Differential MicroRNA Analyses of Burkholderia pseudomallei- and Francisella tularensis-Exposed hPBMCs Reveal Potential Biomarkers
Source: Int J Genomics. 2017 Jul 16;2017:6489383. doi: 10.1155/2017/6489383 (PMC5534298; doi:10.1155/2017/6489383)
Supplement: Supplementary file 1 — Supplementary Figure 1 | Detailed steps used for processing FASTQ files generated from MiSeq runs. Artificially introduced 3' adapter sequences were trimmed, and post-trimmed reads that were a minimum of 15 base pairs were filtered against contaminants. Reads that did not match to contaminants were screened for mature miRNA species (black box) which were further analyzed for statistical significance. Supplementary Figure 2 | RNA purity and quality. (A) The microRNA is seen between 145 nt and 160 bp on a 6% Norvex gel. The ladders used are TruSeq High Resolution Ladder and Custom Ladder (B) Agilent trace shows a good range of small RNA from 145 nt-160 nt after ligation (C) Transcript coverage shows the majority of transcript ranges between 20 and 30 bp. Supplementary Figure 3 | FASTQC analysis. An overview of the range of quality values across all bases at each position in the FastQ file. For each position, a BoxWhisker type plot is drawn. The y-axis on the graph shows the quality scores and in this run, the quality scores range from 30 to 40. Supplementary Figure 4 | Correlation plots of 2 replicates. (A) in hPBMC alone at time point 60 minutes (B) E. coli-exposed hPBMCs at time point 120 minutes. Table S1 | Correlation value (r) between duplicates. Table S2 | 34 differentially expressed miRNAs in E. coli-exposed hPBMCs RNAseq data. Table S3 | 27 differentially expressed miRNAs in B. pseudomallei-exposed hPBMCs RNAseq data. Table S4 | 26 differentially expressed miRNAs in F. tularensis-exposed hPBMCs RNAseq. Table S5 List of 31 miRNAs included in the miScript miRNA PCR assay. Table S6 | Standard deviations for six housekeeping genes. Table S7 | CT values. Table S8 | Fold regulations of miRNAs in different experiments at different time points. Table S9 | Differentialy expressed miRNAs and the number of genes involved in different GO functions. Table S10 | Proposed new broader category for each biological process GO term. Table S11 | A summary table of biological proc [file 6489383.f1.docx]

Supplementary Material

Differential microRNA analyses of *Burkholderia pseudomallei*- and *Francisella tularensis*-exposed hPBMCs reveal potential biomarkers

Regina Z. Cer^1,2*^, J. Enrique Herrera-Galeano^1,2γ^, Kenneth G. Frey^1,2^, Kevin L. Schully^1,2^, Truong V. Luu^1,2^, John Pesce^1,2,§^, Vishwesh P. Mokashi^1±^, Andrea M. Keane-Myers^1,2,†^, Kimberly A. Bishop-Lilly^1,2^

*** Correspondence** Regina Cer: Regina.Z.Cer.ctr@mail.mil

^1^Genomics and Bioinformatics Department, Naval Medical Research Center, Biological Defense Research Directorate, Fort Detrick, MD, USA

^2^Henry M. Jackson Foundation for the Advancement of Military Medicine, Bethesda, MD, USA

***Correspondence**: Regina Cer

[Regina.Z.Cer.ctr@mail.mil](mailto:Regina.Z.Cer.ctr@mail.mil)

**Current address:**

**^γ^**KCE services and consulting, LLC, Columbia MD, USA

§Division of Microbiology and Infectious Diseases, National Institute of Allergy and Infectious Diseases, Bethesda, MD, USA

†Immunology, National Institute of Health, Bethesda, MD, USA

**^±^**Navy Drug Screening Laboratory, Jacksonville, FL, USA

Supplementary Equations

**Creation of Time Series**

Averaged raw sequence count (avgRSC) of each miRNA from hPBMCs alone at t0, referred to as Pt_0_, was subtracted from that at three later time points, 30 min (Pt_30_), 60 min (Pt_60_), and 120 min (Pt_120_ ) (Eq. 1).

$Ptxt0=Ptx-Pt0$ (1)

where:

Pt_0_=avgRSC of miRNAs in hPBMCs alone at t0

Pt_x_=avgRSC of miRNAs in hPBMCs alone at t_x_ where x=30, 60, or 120

After Pt_30_t_0_, Pt_60_t_0_ and Pt_120_t_0_ were calculated, these values were subtracted again from bacterial exposed hPBMCs for the corresponding time points (Eq. 2).

$Otxts=Otx-Ptxt0$ (2)

where:

O=any of the three organisms, *E. coli*, *B. pseudomallei* or *F. tularensis*

Ot_x_=avgRSC of miRNAs in hPBMCs exposed with bacteria O at t_x_ where x=30, 60, or 120

Pt_x_t_0_= obtained in Eq. 1

For example, in the case of *B. pseudomallei*-exposed hPBMCs at time point 60 min (t_60_), the calculations would be Bpt_60_t_s_=Bpt_60_-Pt_60_t_0_ where Bp= *B. pseudomallei* and Pt_60_t_0_ = Pt_60_-Pt_0_.

**qPCR confirmatory analysis**

For each organism, the fold regulations were obtained between hPBMCs alone versus bacteria-exposed hPBMCs for each time point. For example, fold regulations between hPBMCs alone vs. *F. tularensis*-exposed hPBMCs at t30 (Eq. 3), t60 (Eq. 4), and t120 (Eq. 5) were collected.

$x=Pt30 vs.Ot30$ (3)

$y=Pt60 vs.Ot60$ (4)

$z=Pt120 vs.Ot120$ (5)

Raw C_t_ values were converted to fold regulations, and data were collected for each experiment at each time point for each organism in the format miRNA = x, y, z where x, y, z are fold regulations calculated from Equations 3, 4, and 5. To perform the appropriate statistical test, a sign base log transformation (SBLT) that involves the integration of the 2^x^ function producing a function of 1/log dx was implemented. That is, after log transformation was applied to data, they were multiplied by a vector that preserved the numerical sign of the original fold regulation, as shown in Equations 6 and 7.

$\vec{\boldsymbol{d}}=\{30,60,120\}$ (6)

$t=\left( \int_{0}^{\infty} g(|\vec{\boldsymbol{d}}|) \right)* \vec{S}$ (7)

where $\vec{S}=f(\vec{\boldsymbol{d}})$, g(x) = 2^x^ and f(x)=1 if x>0, -1 if x<0.

Supplementary Figures

**
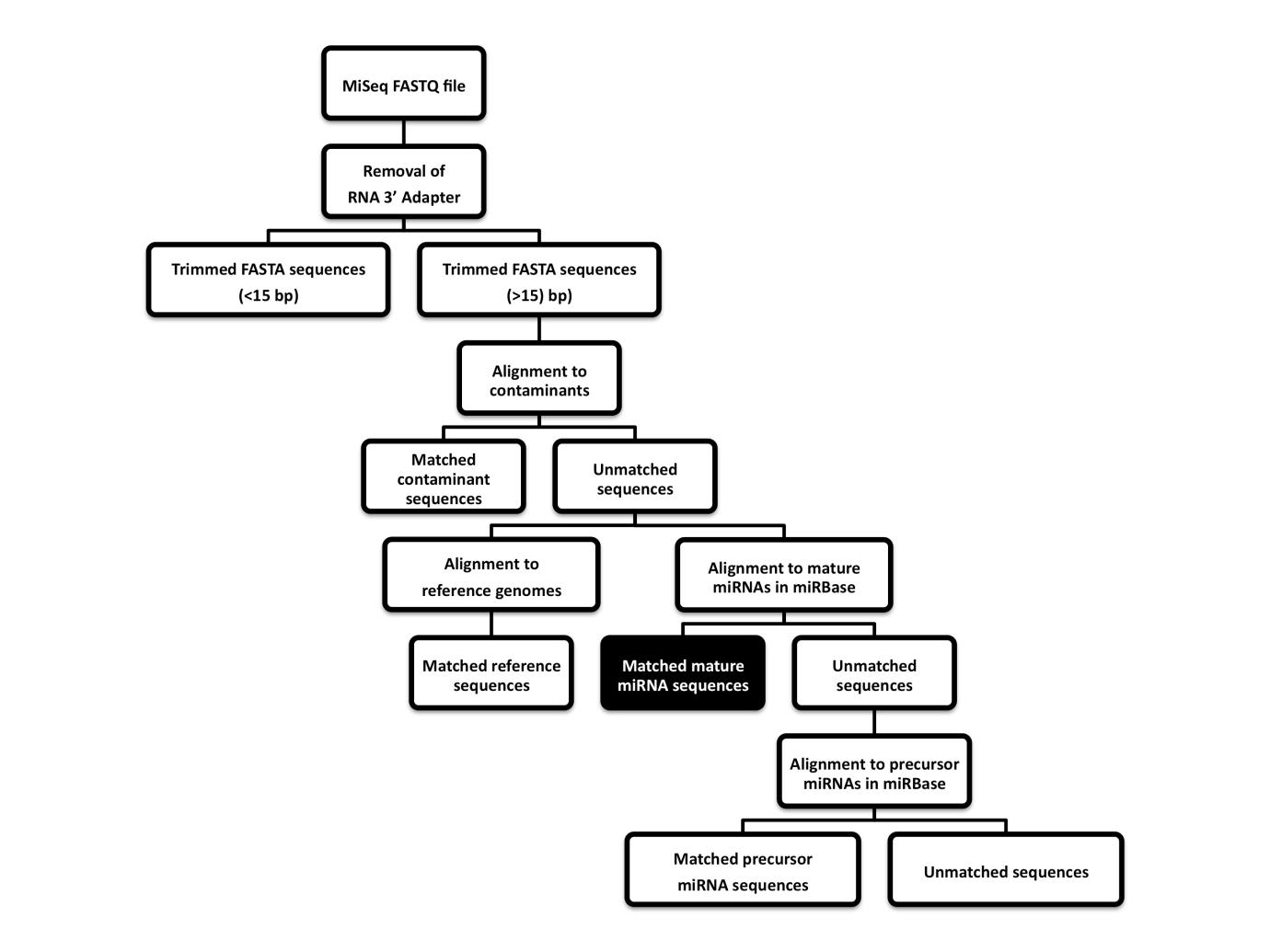
**

**Supplementary Figure 1 | Detailed steps used for processing FASTQ files generated from MiSeq runs.** Artificially introduced 3’ adapter sequences were trimmed, and post-trimmed reads that were a minimum of 15 base pairs were filtered against contaminants. Reads that did not match to contaminants were screened for mature miRNA species (black box) which were further analyzed for statistical significance.

**
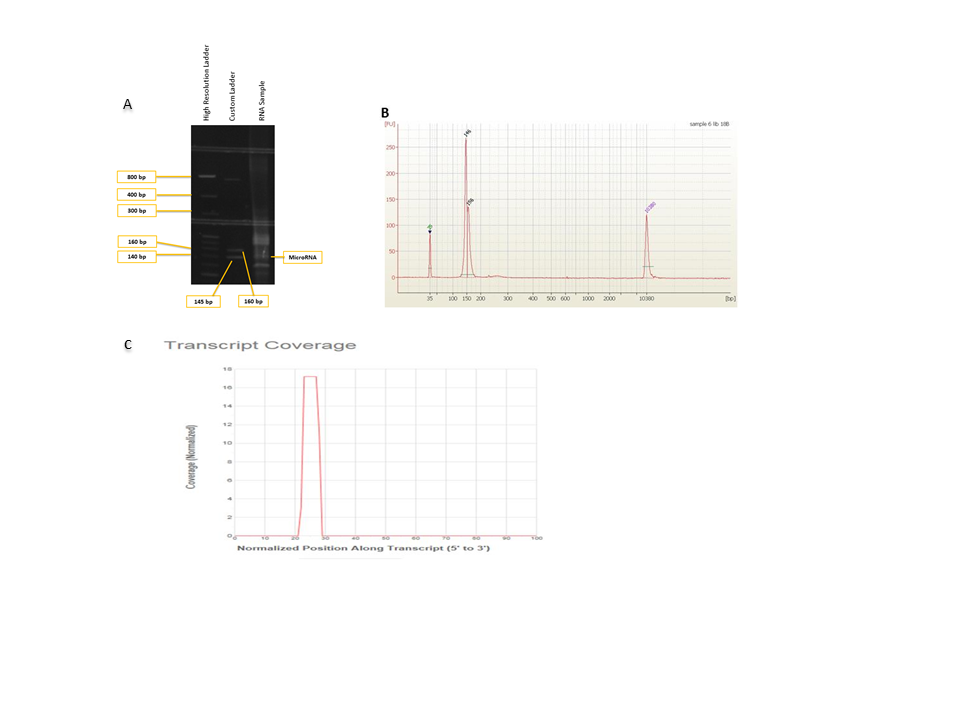
**

**Supplementary Figure 2 | RNA purity and quality.** (**A)** The microRNA is seen between 145 nt and 160 bp on a 6% Norvex gel. The ladders used are TruSeq High Resolution Ladder and Custom Ladder **(B)** Agilent trace shows a good range of small RNA from 145 nt-160 nt after ligation **(C)** Transcript coverage shows the majority of transcript ranges between 20 and 30 bp

**
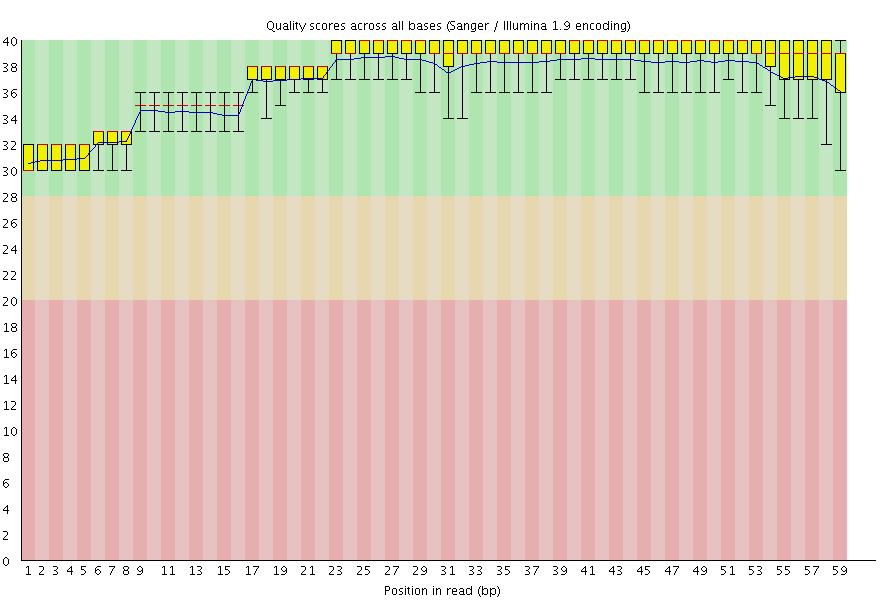
**

**Supplementary Figure 3 | FASTQC analysis.** An overview of the range of quality values across all bases at each position in the FastQ file. For each position, a BoxWhisker type plot is drawn. The y-axis on the graph shows the quality scores and in this run, the quality scores range from 30 to 40.

**
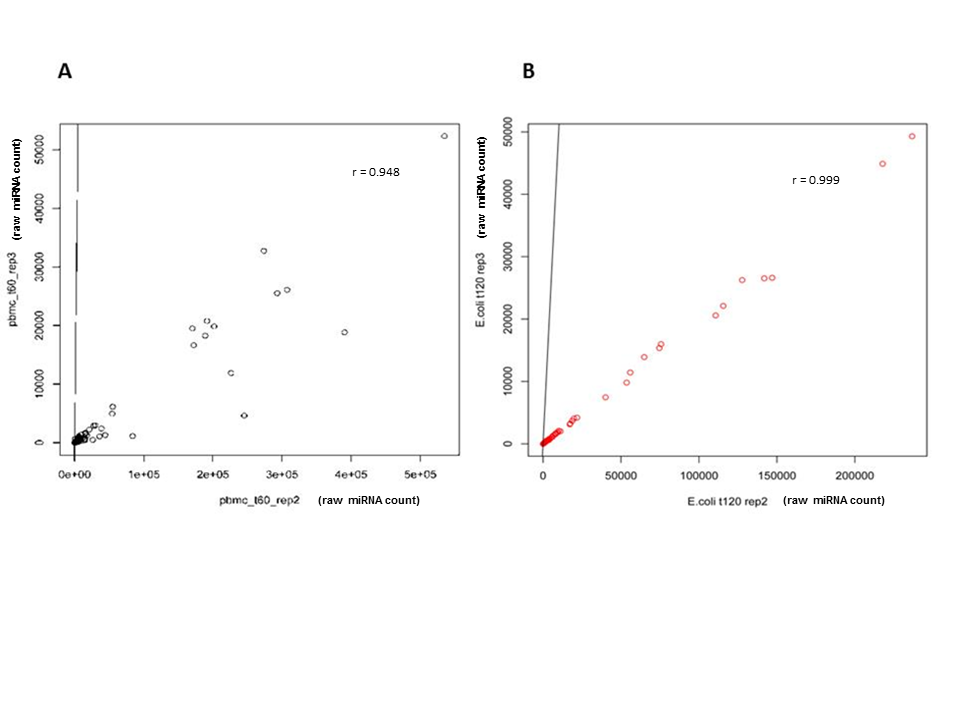
**

**Supplementary Figure 4 | Correlation plots of 2 replicates. (A)** in hPBMC alone at time point 60 minutes **(B)** *E. coli*-exposed hPBMCs at time point 120 minutes
