# Supplementary material for: Differential MicroRNA Analyses of Burkholderia pseudomallei- and Francisella tularensis-Exposed hPBMCs Reveal Potential Biomarkers
Source: Int J Genomics. 2017 Jul 16;2017:6489383. doi: 10.1155/2017/6489383 (PMC5534298; doi:10.1155/2017/6489383)
Supplement: Supplementary file 4 [file 6489383.f4.pptx]

## Slide 1
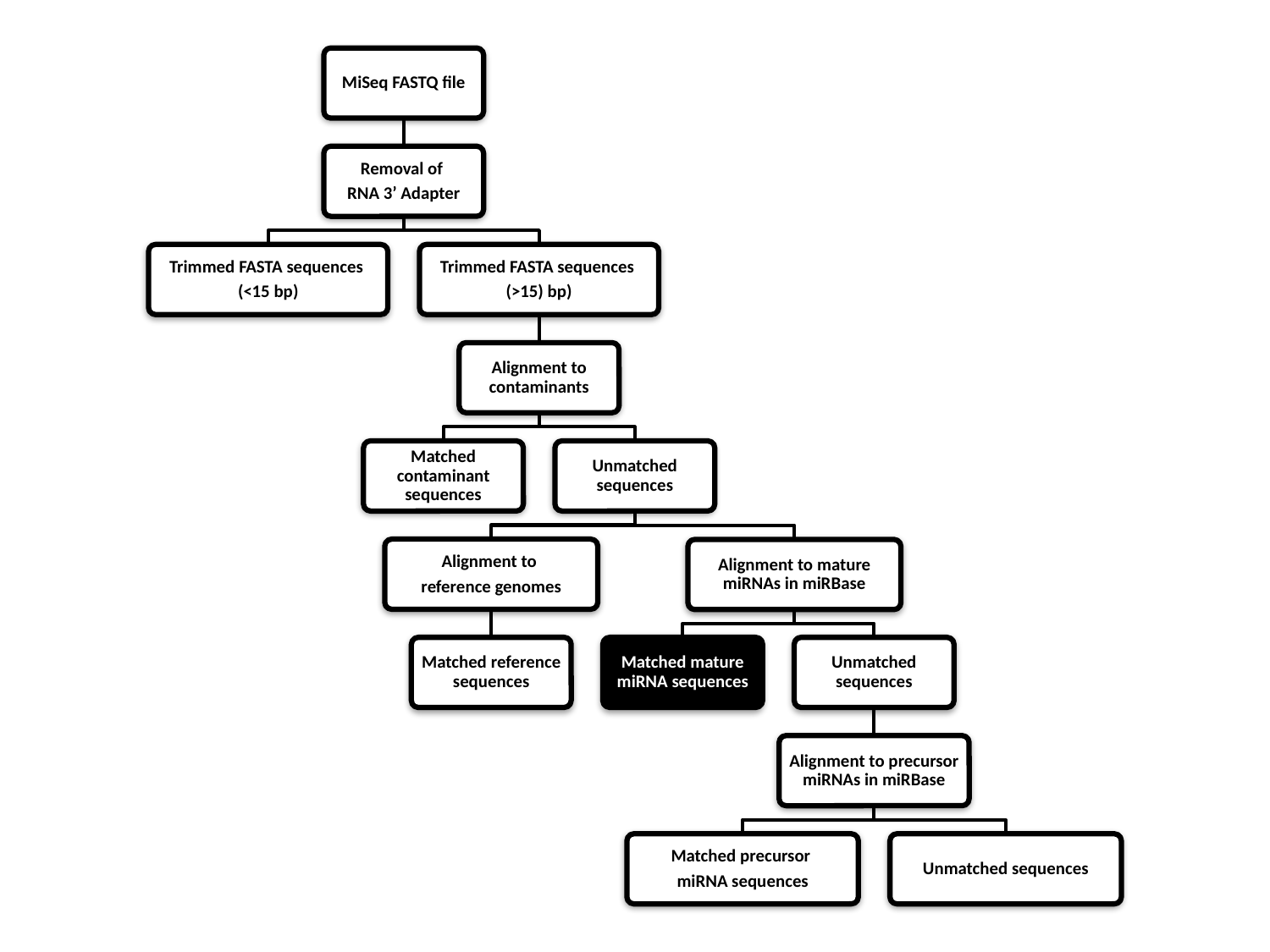

MiSeq FASTQ file
Removal of
RNA 3’ Adapter
Trimmed FASTA sequences
(<15 bp)
Trimmed FASTA sequences
(>15) bp)
Alignment to contaminants
Matched contaminant sequences
Unmatched sequences
Alignment to
reference genomes
Alignment to mature miRNAs in miRBase
Matched reference sequences
Matched mature miRNA sequences
Unmatched sequences
Alignment to precursor miRNAs in miRBase
Matched precursor
miRNA sequences
Unmatched sequences
