# Supplementary material for: Differential MicroRNA Analyses of Burkholderia pseudomallei- and Francisella tularensis-Exposed hPBMCs Reveal Potential Biomarkers
Source: Int J Genomics. 2017 Jul 16;2017:6489383. doi: 10.1155/2017/6489383 (PMC5534298; doi:10.1155/2017/6489383)
Supplement: Supplementary file 5 [file 6489383.f5.pptx]

## Slide 1
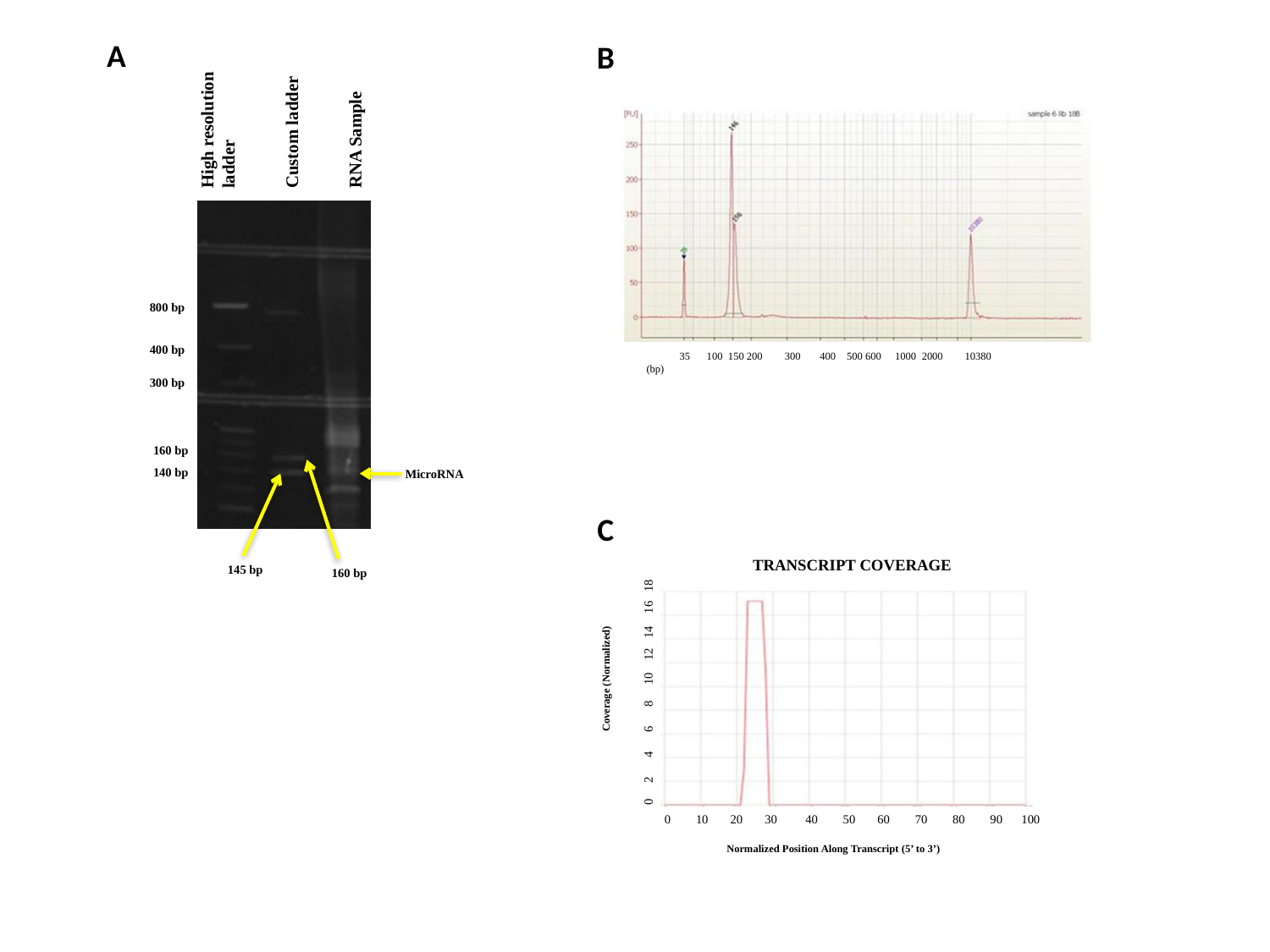

High resolution ladder
Custom ladder
RNA Sample
A
B
800 bp
400 bp
 35 100 150 200 300 400 500 600 1000 2000 10380 (bp)
300 bp
160 bp
140 bp
MicroRNA
C
TRANSCRIPT COVERAGE
145 bp
160 bp
 0 2 4 6 8 10 12 14 16 18
Coverage (Normalized)
0 10 20 30 40 50 60 70 80 90 100
Normalized Position Along Transcript (5’ to 3’)
