# Supplementary figures and images for: Differential MicroRNA Analyses of Burkholderia pseudomallei- and Francisella tularensis-Exposed hPBMCs Reveal Potential Biomarkers
Source: Int J Genomics. 2017 Jul 16;2017:6489383. doi: 10.1155/2017/6489383 (PMC5534298; doi:10.1155/2017/6489383)

## Slide 1
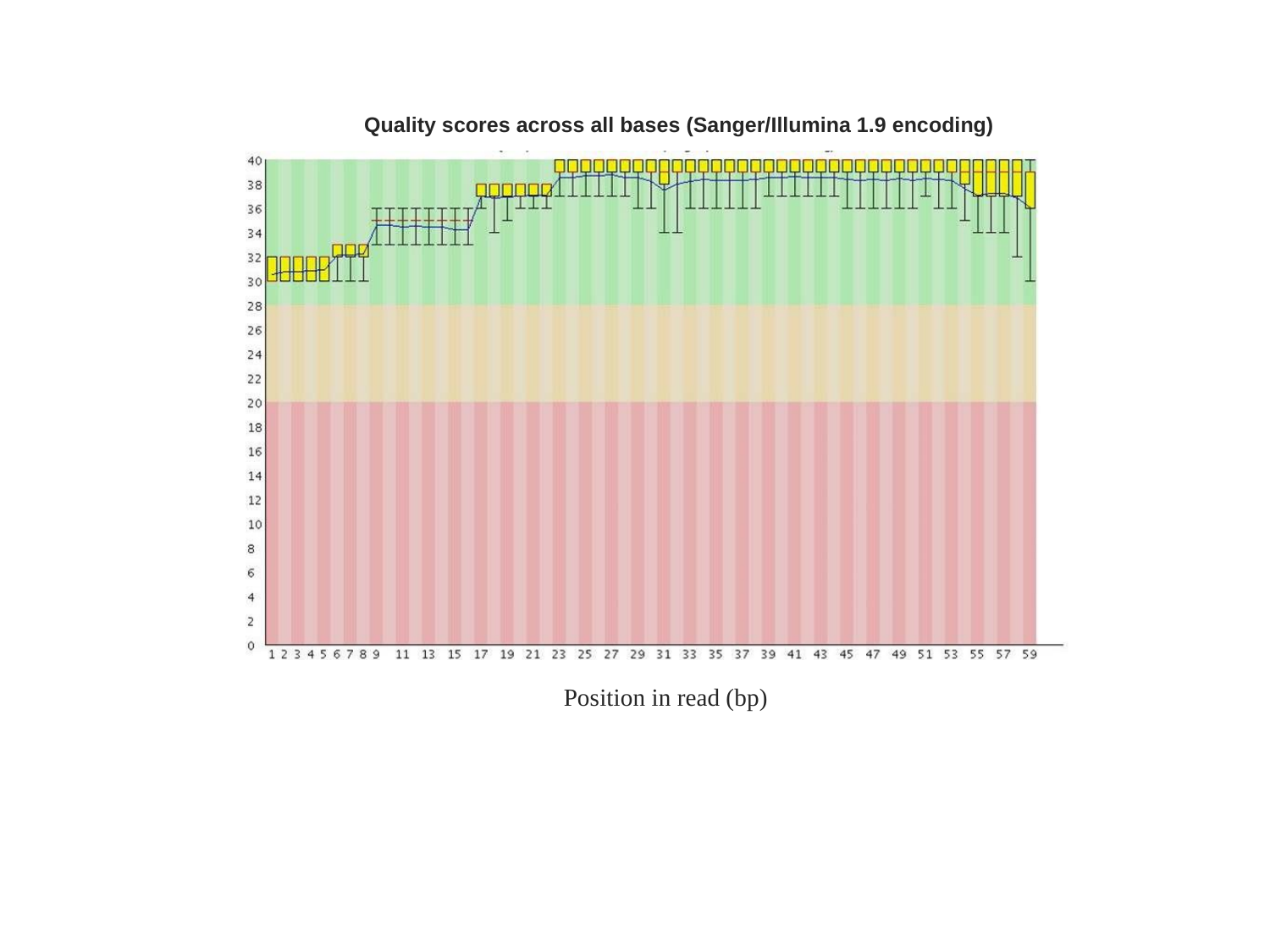

Quality scores across all bases (Sanger/Illumina 1.9 encoding)
Position in read (bp)

Supplement: Supplementary file 6 [file 6489383.f6.pptx]
