# Supplementary material for: Differential MicroRNA Analyses of Burkholderia pseudomallei- and Francisella tularensis-Exposed hPBMCs Reveal Potential Biomarkers
Source: Int J Genomics. 2017 Jul 16;2017:6489383. doi: 10.1155/2017/6489383 (PMC5534298; doi:10.1155/2017/6489383)
Supplement: Supplementary file 7 [file 6489383.f7.pptx]

## Slide 1
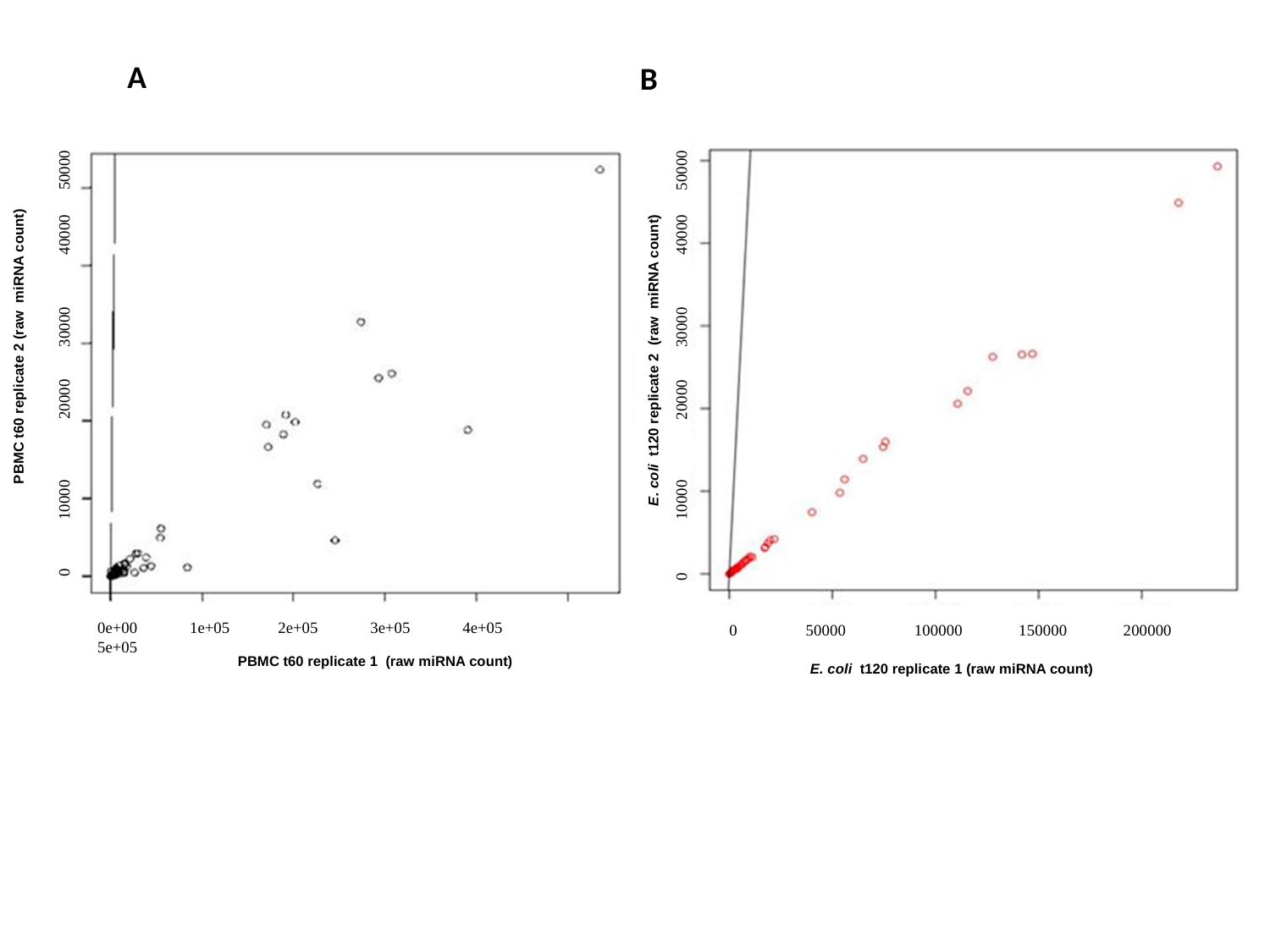

A
B
0 10000 20000 30000 40000 50000
 0 10000 20000 30000 40000 50000
PBMC t60 replicate 2 (raw miRNA count)
 E. coli t120 replicate 2 (raw miRNA count)
0e+00 1e+05 2e+05 3e+05 4e+05 5e+05
0 50000 100000 150000 200000
PBMC t60 replicate 1 (raw miRNA count)
E. coli t120 replicate 1 (raw miRNA count)
